# Sex-specific differences in salivary microbiota composition and their associations with metabolic health in adults with excess body weight: a secondary cross-sectional analysis

**DOI:** 10.1007/s00784-026-06826-7

**Published:** 2026-03-23

**Authors:** Jhenifer Pereira da Costa, Gabriela Macedo Fraiz, Dandara Baia Bonifácio, Fermín I. Milagro, Josefina Bressan

**Affiliations:** 1 Department of Nutrition and Health, Universidade Federal de Viçosa, Viçosa, Minas Gerais 36570-900 Brazil; 2 Department of Nutrition, Food Science and Physiology, Centre for Nutrition Research, Universidad de Navarra, Pamplona, Navarra 31008 Spain; 3 Centro de Investigación Biomédica en Red de la Fisiopatología de la Obesidad y Nutrición (CIBERobn), Institute of Health Carlos III (Instituto de Salud Carlos III), Madrid, Comunidad de Madrid 28029 Spain; 4 Navarra Institute for Health Research (IdiSNA), Pamplona, Navarra 31008 Spain

**Keywords:** Obesity, Microbial Community, Saliva, Sex characteristics, Cardiometabolic Risk Factor

## Abstract

**Objectives:**

Growing evidence links the oral microbiome to obesity-related outcomes, yet the influence of sex-related biological differences on salivary microbial profiles remains insufficiently explored. This exploratory study aimed to characterize the composition, abundance, and diversity of the salivary microbiota in Brazilian men and women with excess body weight and to examine its associations with cardiometabolic markers.

**Materials and methods:**

This cross-sectional secondary analysis of a randomized controlled trial (REBEC: RBR-9832wsx) included 59 adults with excess body weight. Salivary microbiota was profiled through amplification of the 16 S rRNA V4 region, and bioinformatics analyses were performed using the Microbiome Analyst (v2.0). Associations between microbial taxa and clinical variables were assessed using Spearman’s correlation.

**Results:**

Men exhibited greater alpha diversity at the family level by higher Shannon (*p* = 0.015, r_rb_ = 0.4) and Simpson (*p* = 0.003, r_rb_ = 0.5) indices. Sex-specific microbial differences were identified: men showed higher levels of genera *Tannerella*, *Lachnoanaerobaculum*, and *Actinomyces*, as well as the putative species-level taxons *Tannerella serpentiformis* and *Lachnoanaerobaculum umeaense*, whereas women demonstrated greater abundance of genera *Campylobacter*, *Granulicatella*, *Moryella*, and *Scardovia*. Among women, *Granulicatella* genera was positively associated with triglycerides and the TyG index.

**Conclusion:**

Men and women with excess body weight exhibited distinct salivary microbiota profiles, with differences in both diversity and taxonomic composition.

**Clinical relevance:**

Sex-specific differences in salivary microbiota composition may be associated with variations in metabolic markers. These findings are exploratory and hypothesis-generating, providing insight into sex-related patterns in the oral microbiome and may help inform future research exploring personalized approaches to cardiometabolic risk assessment.

## Introduction

Overweight and obesity are characterized by the excessive body fat accumulation, negatively affecting health¹. This condition has social, economic, and psychological consequences, impacting a large portion of the population and becoming a global epidemic [1]. According to the World Obesity Atlas 2025, at least half of the world’s adult population will be overweight by 2030 [2]. In Brazil, the 2023 VIGITEL survey showed that 1 in 4 adults has obesity (24.8% women; 23.8% men) [3]. Obesity’s pro-inflammatory state and increased oxidative stress are associated with chronic diseases, such as type 2 diabetes and cardiovascular diseases [1, 2]. It is also linked to gut and oral dysbiosis, characterized by lower microbial diversity and higher abundance of pathogenic bacteria [4].

The oral cavity is the second-largest microbial reservoir in the human body, housing about 770 bacterial species, around 120 of which are shared with the gut [5]. Its composition is influenced by genetics, age, lifestyle, diet, oral hygiene, and oral diseases [5]. Oral conditions like periodontitis and cavities can alter the bacterial communities and are related to metabolic imbalances, endotoxemia, and inflammation [5–8]. Moreover, gut and oral dysbiosis are associated with increased prevalence of excess body weight [4, 9].

Two main pathways connect salivary microbiota to obesity: the enteral route – via swallowed bacteria resistant to gastric acidity (e.g.,*Streptococcus* spp., *Porphyromonas gingivalis*,* Klebsiella*,* Helicobacter pylori*,* Vellonella*,* Parvimonas micra*,* Fusobacterium nucleatum*) – and the hematogenous route, where bacteria enter the bloodstream through oral lesions and reach the intestinal mucosa and other organs [5, 6, 9–11].

Despite increasing evidence connecting oral microbiota and obesity, sex-based differences remain poorly understood, particularly in populations with excess body weight [12–14]. Studies on gut microbiota have already shown that sex hormones, fat distribution, and immune profiles modulate microbial diversity and composition, leading to different metabolic responses between men and women [15, 16]. However, whether similar sex-related patterns occur in the salivary microbiota and how these might relate to cardiometabolic health remains largely unexplored.

Understanding sexual dimorphism in salivary microbiota may help identify sex-specific microbial signatures associated with cardiometabolic risk, contributing to the development of personalized nutrition and preventive strategies. Such knowledge may guide the design of more precise interventions for obesity management, integrating oral and metabolic health. Therefore, this study aimed to characterize the composition, abundance and diversity of the salivary microbiota in individuals with excess body weight, according to sex and to explore its implications for cardiovascular and metabolic health.

## Methods

### Recruitment of participants and experimental design

This study was a secondary analysis of a randomized controlled clinical trial lasting ten weeks, originally designed to evaluate the effect of green tea kombucha on the metabolic health of individuals with excess body weight. Details regarding the methodology, sample size calculation and results of the primary intervention have been previously described [17]. For this study, baseline data from 59 participants were collected.

Briefly, individuals aged 18–45 years were recruited based on the inclusion criteria: BMI ≥ 27 kg/m², waist circumference (WC) ≥ 80 cm for women and ≥ 94 cm for men, and body fat (BF) > 30% for women and > 25% for men [18]. Among the non-inclusion criteria, the participants could not present other inflammatory or metabolic disorders, regular use of nutritional supplements, antibiotics, anti-inflammatory medications, or any other that affects lipid and/or glucose metabolism; smokers, pregnant and/or breastfeeding women.

### Ethical aspects

The project was submitted to the Ethics Committee for Research Involving Human Beings at the Federal University of Viçosa (UFV) (CAAE: 25880819.3.0000.5153; report number: 3.948.033). The procedures described were established in accordance with the Resolution CNS/466 of 2012 and the Helsinki Declaration. The project was also registered in the Brazilian Clinical Trials Registry (REBEC) (number: RBR-9832wsx). All participants signed the Informed Consent Form (ICF).

### Assessments and measurements

Sociodemographic data were collected through a structured questionnaire containing information on education level, income, and sex.

Dietary intake was evaluated through food frequency questionnaires (FFQ), which were administered by trained nutritionists to assess the individual’s usual food intake during the intervention. All dietary consumption data were converted into mass (grams) and/or volume (milliliters) using the ERICA-REC24h (version 24_05_2022) [19], a software that allows the input of frequency-based dietary data and estimates nutrient intake based on Brazilian food composition tables developed by the Brazilian Institute of Geography and Statistics (IBGE). When a food or food preparation was not included in this database, the Brazilian Food Composition Table (TBCA/USP) [20] was used.

The assessment of anthropometric parameters was conducted at the Laboratory of energy Metabolism and Body Composition (LAMECC). Participants were weighed on a digital electronic scale (InBody^®^, model 230, BiospaceCo). Height was measured with a vertical stadiometer in millimeters fixed to the wall (SECA^®^, mosel 206, Hamburg, Germany). Hip circumference (HC) and WC were measured with a flexible, non-elastic measuring tape, following specific protocols [21]. Body composition was analyzed using Dual-energy X-ray Absorptiometry (DEXA) at the Health Division of UFV, total fat mass, lean mass, and fat mass from the android, gynoid, and trunk regions were considered. Furthermore, blood pressure was measured using the automatic Omron^®^ model HEM-7113 device [22].

Blood samples were collected and analyzed by qualified nursing technicians at the Clinical Analysis Laboratory of the UFV Health Division (LACDSA). Among the biochemical determinations considered are: total cholesterol (TC), high-density lipoprotein (HDL), low-density lipoprotein (LDL), triglycerides (TG), fasting blood glucose (FBG), insulin, C-reactive protein (CRP), interleukin 6 (IL-6) and interleukin 8 (IL-8).

The anthropometric parameters and cardiometabolic markers were considered for characterizing the sample and for subsequent association with the diversity parameters (alpha and beta) of the microbiota.

### Saliva collection and microbiota analysis

Unstimulated saliva samples were collected by obtaining 5 mL of spontaneous saliva in a sterile container. Participants were instructed to refrain from food and beverages for at least eight hours prior to collection. After collection, the saliva was separated and stored at -80 °C (Thermo Scientific/Forma 900 Series^®^) until subsequent analyses. Genomic DNA extraction was conducted at the Experimental Nutrition Laboratory of the Department of Nutrition and Health at UFV, Brazil, using the QIAamp^®^ DNA Mini Kit (Qiagen, Hilden, Germany) according to the manufacturer’s protocol. The purity of the DNA was determined by the A260/A280 ratio, using a NanoDrop 7000 Spectrophotometer (Thermo Fisher Scientific, Waltham, MA, USA), while the integrity of the DNA was verified through 1% agarose gel electrophoresis. After extraction and purification, the DNA was sent by mail to the University of Illinois, Chicago, to the Genome Research Core, where amplicon preparation and sequencing took place. PCR amplification of the V4 region of the prokaryotic 16 S rRNA gene was performed with Next Generation Sequencing (NGS), using universal primers (samples: 16 S-U515F and 16 S-U806R). All PCR products were sequenced on an Illumina NovaSeq6000 instrument, following standard protocols (Illumina, Inc., San Diego, CA, USA) [17].

The 16 S rRNA gene sequencing data were processed using the Quantitative Insights Into Microbial Ecology platform (QIIME2, v.2020.8; http://www.qiime2.org) for raw sequence analysis (doi: 10.1038/s41587-019-0209-9). Paired-end reads were trimmed using the *cutadapt* plugin in QIIME2 (doi: 10.14806/ej.17.1.200). The sequences were then merged, filtered to remove low-quality reads and chimeric sequences, and then assigned to amplicon sequence variants (ASVs) using the *dada2* plugin in QIIME2 (doi:10.1038/nmeth.3869). Taxonomic assignment was performed using the Ribosomal Database Project (RDP) naïve Bayesian classifier (Version 2.14), trained on the V4 region of the 16 S rRNA gene corresponding to the primer pair 16 S-U515F and 16 S-U806R. Taxonomic confidence was assessed using default QIIME2 parameters (confidence threshold = 0.7). Species-level assignments were interpreted cautiously and are reported as putative species-level classifications. The RDP database was selected due to its robust curation, broad taxonomic coverage, and frequent application in microbiome studies, allowing reliable classification across multiple taxonomic levels.

Negative controls (non-template controls, NTCs) were included during sequencing and used to assess potential contamination. After sequence merging, quality and length filtering, primer removal, and ASV inference using DADA2, contaminant detection was performed using the *decontam* package based on prevalence in NTC samples. This procedure identified 18 contaminant ASVs, which were removed prior to downstream analyses. In addition, ASVs corresponding to known laboratory contaminants, including *Mycoplasma*-associated taxa, were manually excluded when not fully captured by automated detection. Host-associated sequences, such as chloroplast and mitochondrial ASVs, were also removed from the dataset. Sequencing generated a mean depth of approximately 120,000 reads per sample.

The alpha diversity assessment was performed using the Chao 1, Shannon, and Simpson indices [23] from non-rarefied feature tables, considering the Mann-Whitney U test for comparisons between men and women. Effect sizes were reported for diversity indices showing statistically significant between-group differences using rank-biseral correlation (r_rb_) through Jamovi 2.7.17 program. For the analysis of beta diversity, the Bray-Curtis dissimilarity index was calculated, using PERMANOVA for statistical significance, and homogeneity of multivariate dispersion was evaluated using PERMDISP to assess whether observed patterns were influenced by heterogeneous within-group variability. For differential abundance analysis, the identification of discriminant taxa between groups was performed by transforming the counts by the centralized log-ratio (CLR) and applying ALDEx2 for all taxonomic levels. Differential abundance analyses using ALDEx2, which explicitly accounts for data compositionality and reduces false-positive findings, were conducted to directly compare taxa between men and women, independently of global beta-diversity patterns. In addition, for the correction of multiple tests, the Benjamini-Hochberg approach was applied to control the false discovery rate (FDR), significance was defined with a threshold of 0.05 for the FDR-corrected p-values. Associations between CLR-transformed taxon abundances and cardiometabolic variables were evaluated using Spearman’s rank correlation. Given the exploratory nature of this secondary cross-sectional analysis, an FDR threshold of 0.10 was considered for correlation analyses, while unadjusted p-values < 0.05 were reported as nominal associations.

### Statistical analysis

The Shapiro-Wilk test, histograms and box plots were used to verify the normality of quantitative variables. These were presented as mean (standard deviation) or median (25th -75th percentile), depending on the adherence to the normal distribution of the variables. Qualitative variables were presented in absolute and relative frequency. The Student’s t-test (or Mann-Whitney test) was used to assess differences between men and women for continuous variables, while the chi-square test was used for categorical variable analysis. The database was created using Microsoft Office Excel version 16.49, 2021©. The analyses were conducted using SPSS software (IBM Corp. Released in 2017. IBM SPSS Statistics for Windows, Version 25.0. Armonk, NY: IBM Corp.). A statistical significance level (α) of 0.05 was adopted for the analyses.

## Results

Some statistical differences were observed between men and women. Male participants had greater weight, waist circumference, and muscle mass compared to female participants, while female participants had greater total body fat and gynoid fat (Table 1). Regarding dietary consumption and sociodemographic characteristics, no statistically significant differences were found between the sexes (Table 1).

**Table 1:** Sociodemographic, anthropometric, cardiometabolic characteristics, and dietary consumption according to sex

	Participants (*n* = 59)	Women (*n* = 35)	Men (*n* = 24)	*p* value
Sociodemographic characteristics				
Age, years	33.61 (6.95)	33.23 (6.77)	34.17 (7.23)	0.615
Race, n (%)				
white	26 (44.1)	16 (45.7)	10 (41.7)	0.618
mixed race	23 (39.0)	13 (37.1)	10 (41.7)	
black	9 (15.3)	6 (17.1)	3 (12.5)	
indigenous	1 (1.7)	-	1 (4.2)	
Education, n (%)				
elementary/high school	7 (11.9)	6 (17.1)	1 (4.2)	0.326
incomplete higher education	17 (28.8)	9 (25.7)	8 (33.3)	
complete higher education	35 (59.3)	20 (57.1)	15 (62.5)	
*Income, n (%)				
up to 3 minimum wages	17 (28.8)	14 (40.0)	3 (12.5)	0.084
3 to 5 minimum wages	15 (25.4)	6 (17.1)	9 (37.5)	
5 to 10 minimum wages	21 (35.6)	13 (37.1)	8 (33.3)	
> 10 minimum wages	6 (10.2)	2 (5.7)	4 (16.7)	
Marital status, n (%)				
single	29 (49.2)	20 (57.1)	9 (37.5)	0.333
married	28 (47.5)	14 (40.0)	14 (58.3)	
divorced	2 (3.4)	1 (2.9)	1 (4.2)	
Anthropometric and body composition parameters				
Weight, kg	92.01 (13.36)	87.52 (13.07)	98.55 (11.07)	**0.001**
BMI, kg/m²	32.75 (3.71)	33.32 (4.17)	31.92 (2.78)	0.154
Total body fat, kg	39.91 (8.11)	42.35 (8.55)	36.37 (5.97)	**0.004**
Gynoid body fat, kg	7.44 (1.68)	8.10 (1.69)	6.47 (1.11)	**< 0.001**
Android body fat, kg	3.31 (0.89)	3.23 (1.01)	3.43 (0.70)	0.410
Total muscle mass, kg	48.52 (10.60)	41.74 (5.60)	58.41 (8.07)	**< 0.001**
Hip circumference, cm	115.12 (8.10)	116.61 (9.14)	112.93 (5.79)	0.087
Waist circumference, cm	95.08 (9.78)	91.54 (9.08)	100.25 (8.51)	**< 0.001**
LAP	51.32 (30.50)	47.97 (27.32)	56.21 (34.65)	0.313
Cardiometabolic markers				
Glucose, mg/dL	86.48 (7.02)	85.71 (7.45)	87.65 (6.28)	0.308
Insulin, mg/dL	10.74 (4.00)	10.53 (4.10)	11.03 (3.93)	0.647
HOMA-IR	2.45 (1.03)	2.48 (1.10)	2.42 (0.95)	0.871
TyG index	4.60 (0.25)	4.58 (0.22)	4.64 (0.29)	0.360
Total cholesterol, mg/dL	181.76 (32.33)	183.74 (30.37)	178.87 (35.45)	0.574
LDL-c, mg/dL	112.93 (26.45)	112.29 (24.94)	113.83 (28.97)	0.829
HDL-c, mg/dL	41.95 (9.65)	45.66 (9.22)	36.54 (7.59)	**< 0.001**
VLDL, mg/dL	26.10 (13.45)	24.46 (10.84)	28.50 (16.51)	0.260
TG, mg/dL	130.36 (67.25)	122.11 (54.24)	142.37 (82.47)	0.431
IL-6	3.96 (3.46)	4.12 (3.41)	3.77 (3.59)	0.729
IL-8	14.28 (18.37)	10.37 (9.03)	19.49 (25.46)	0.066
CRP	0.99 (1.65)	0.99 (1.09)	1.00 (2.26)	0.976
SBP, mm Hg	115.10 (10.62)	111.17 (8.40)	120.83 (11.08)	**< 0.001**
DBP, mm Hg	74.22 (7.59)	73.51 (7.50)	75.25 (7.84)	0.393
Dietary consumption				
Energy, kcal/d	2,041 (645)	1,889 (447)	2,263 (818)	0.081
Carbohydrates, %	46.75 (6.73)	46.51 (5.67)	47.12 (8.16)	0.736
Lipids, %	36.14 (5.86)	36.62 (5.20)	35.44 (6.78)	0.451
Proteins, %	16.49 (2.27)	16.44 (2.33)	16.56 (2.23)	0.834
Fiber, g/d	10.43 (1.99)	10.67 (1.94)	10.05 (2.06)	0.245
Added sugar, g/d	39.33 (23.74)	39.1 (20.7)	39.7 (28.0)	0.927

Quantitative data expressed as mean (SD). P-value: comparison between women and men (independent t-test; significance indicated in bold). *1 minimum wage= R$ 1.412,00. *HDL-c* High-Density Lipoprotein, *HOMA-IR* Homeostasis Model Assessment of Insulin Resistance, *BMI* Body Mass Index, *LAP* Lipid accumulation product, *LDL-c* Low-Density Lipoprotein, *DBP* Diastolic Blood Pressure, *SBP* Systolic Blood Pressure, *TG* Triglycerides, *TyG index* Triglycerides-Glucose Index, *VLDL* Very Low-Density Lipoprotein, *IL-6* Interleukin 6, *IL-8* Interleukin 8, *CRP* C-reactive protein

Significant differences in the bacterial profile abundance were observed between men and women with excess body weight (Table 2). At the species level, men exhibited a significantly higher relative abundance of putative species *Tannerella serpentiformi*s, *Cardiobacterium valvarum*, *Lachnoanaerobaculum umeaense*, *Actinomyces graevenitzii*, and *Stomatobaculum longum*, whereas women showed a significantly higher abundance of *Campylobacter concisus*. The genera *Tannerella*, *Lachnoanaerobaculum*, *Stomatobaculum*, and *Actinomyces* were found in greater abundance in men, while the genera *Campylobacter*, *Pseudostreptobacillus*, *Granulicatella*, *Moryella*, and *Scardovia* were more abundant in women. Regarding the family (Carnobacteriaceae, Campylobacteraceae), class (Campylobacteria, Bacilli), and order (Campylobacterales), the highest abundance was found in women, except for the class Clostridia, which was more abundant in men (Table 2).

**Table 2:** Statistically significant most abundant bacterial taxa between men and women

		Effect	P value	FDR	↑men/women
Species	*Tannerella serpentiformis*	-3.3349	2.0453E-12	2.0453E-10	↑men
	*Cardiobacterium valvarum*	-1.6439	1.8694E-4	0.00727	↑men
	*Lachnoanaerobaculum umeaense*	-1.3991	2.1822E-4	0.00727	↑men
	*Actinomyces graevenitzii*	-1.536	3.574E-4	0.00893	↑men
	*Campylobacter concisus*	-2.4772	6.4217E-4	0.01249	↑women
	*Stomatobaculum longum*	-1.1887	7.499E-4	0.01249	↑men
Genus	*Tannerella*	-2.1912	2.2326E-8	1.1386E-6	↑men
	*Lachnoanaerobaculum*	-1.0816	0.0015526	0.03004	↑men
	*Campylobacter*	-1.3632	0.00197	0.03004	↑women
	*Pseudostreptobacillus*	2.2635	0.0029272	0.03004	↑women
	*Stomatobaculum*	-0.94526	0.0031988	0.03004	↑women
	*Granulicatella*	0.88495	0.0035345	0.03004	↑women
	*Actinomyces*	-1.0755	0.0050156	0.03654	↑men
	*Moryella*	1.6046	0.0073242	0.04222	↑women
	*Scardovia*	1.3101	0.0074508	0.04222	↑women
Family	Campylobacteraceae	-1.3015	0.0016141	0.042336	↑women
	Carnobacteriaceae	0.83227	0.003136	0.042336	↑women
Class	Campylobacteria	-1.2038	0.0042344	0.043911	↑women
	Clostridia	-0.4457	0.0071029	0.043911	↑men
	Bacili	0.671	0.0094094	0.043911	↑women
Order	Campylobacterales	-1.3916	5.6819E-4	0.011364	↑women

↑: higher

**Fig. 1:**
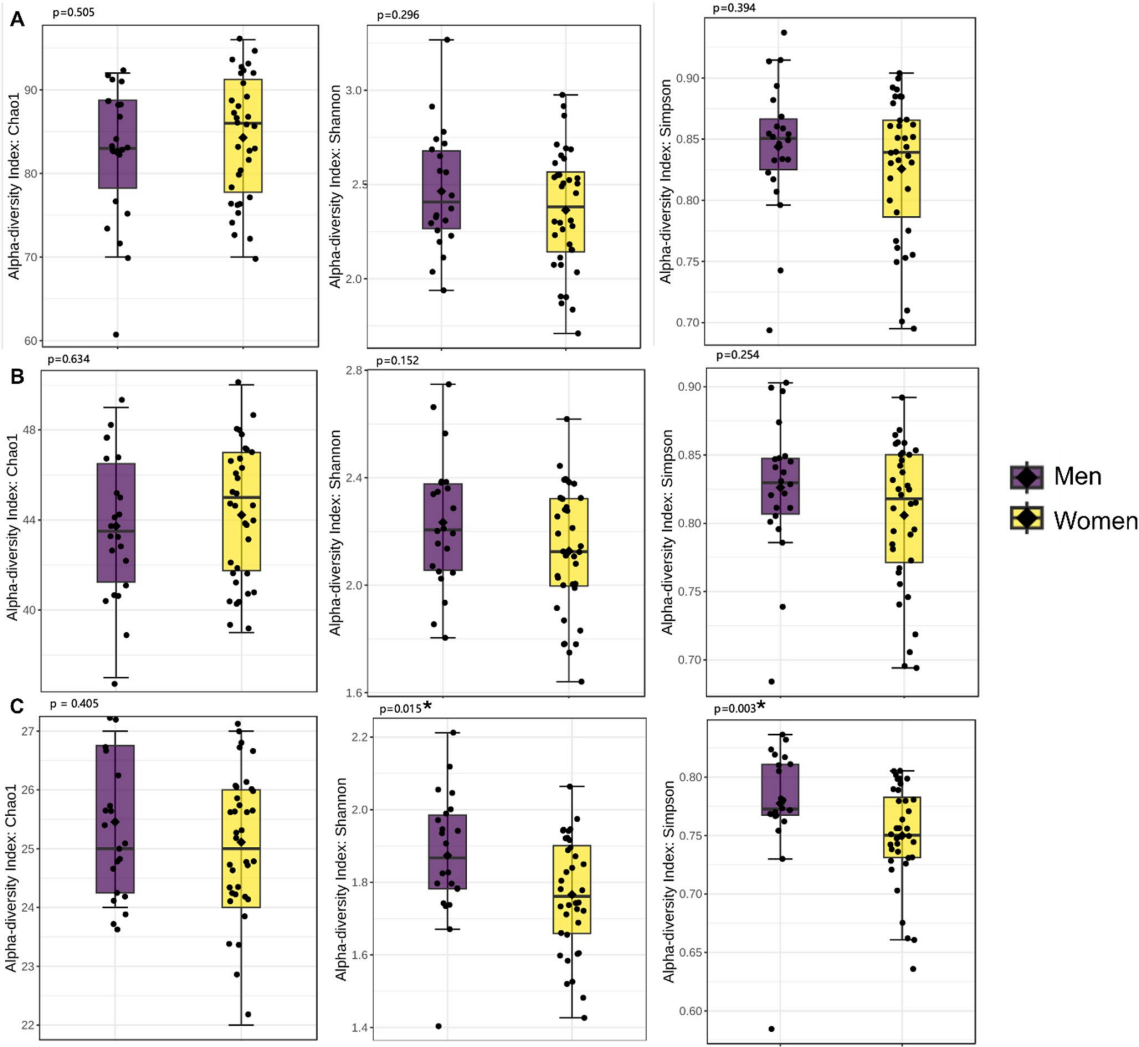
Difference in alpha diversity by Chao1, Shannon, and Simpson at the (**A**) species, (**B**) genus, and (**C**) family levels between men and women with excess body weight (men: purple; women: yellow). The multi-testing adjustment is based on Benjamini-Hochberg procedure procedure (FDR)

Alpha diversity, represented by the Shannon (*p* = 0.015, r_rb_ = 0.4) and Simpson (*p* = 0.003, r_rb_ = 0.5) indices, was higher in males compared to females at the family level (Fig. 1). No other significant differences were observed regarding the other taxonomic levels nor in the Chao 1 richness index (Fig. 1).

**Fig. 2:**
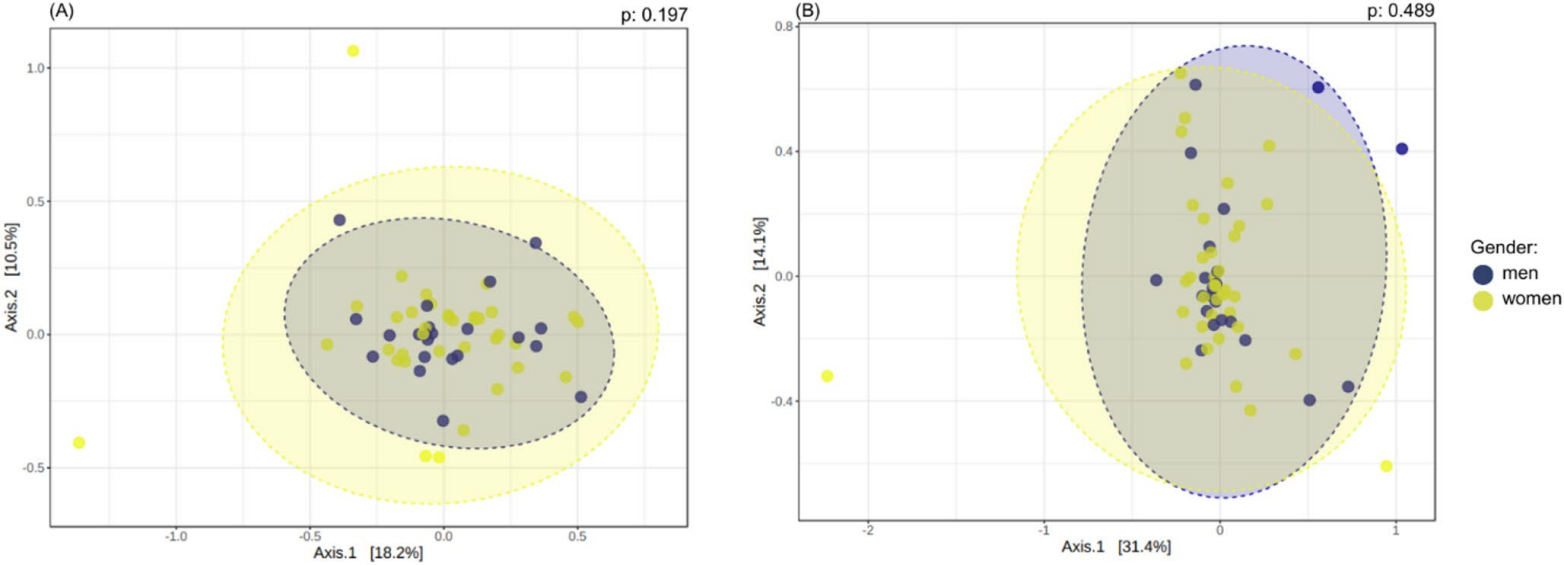
Beta diversity differentiation between men and women according to Bray-Curtis dissimilarity at the (**A**) species level (*p* = 0.197) and (**B**) genus level (*p* = 0.489). Homogeneity of multivariate dispersion was assessed using PERMDISP and did not differ between sexes (*p* > 0.05)

Furthermore, beta diversity assessed by Bray–Curtis dissimilarity did not differ significantly between men and women at either the species or genus level (PERMANOVA, *p* > 0.05; Fig. 2). Homogeneity of dispersion was confirmed by PERMDISP (*p* > 0.05), indicating that the absence of differences was not driven by unequal within-group variability.

**Fig. 3:**
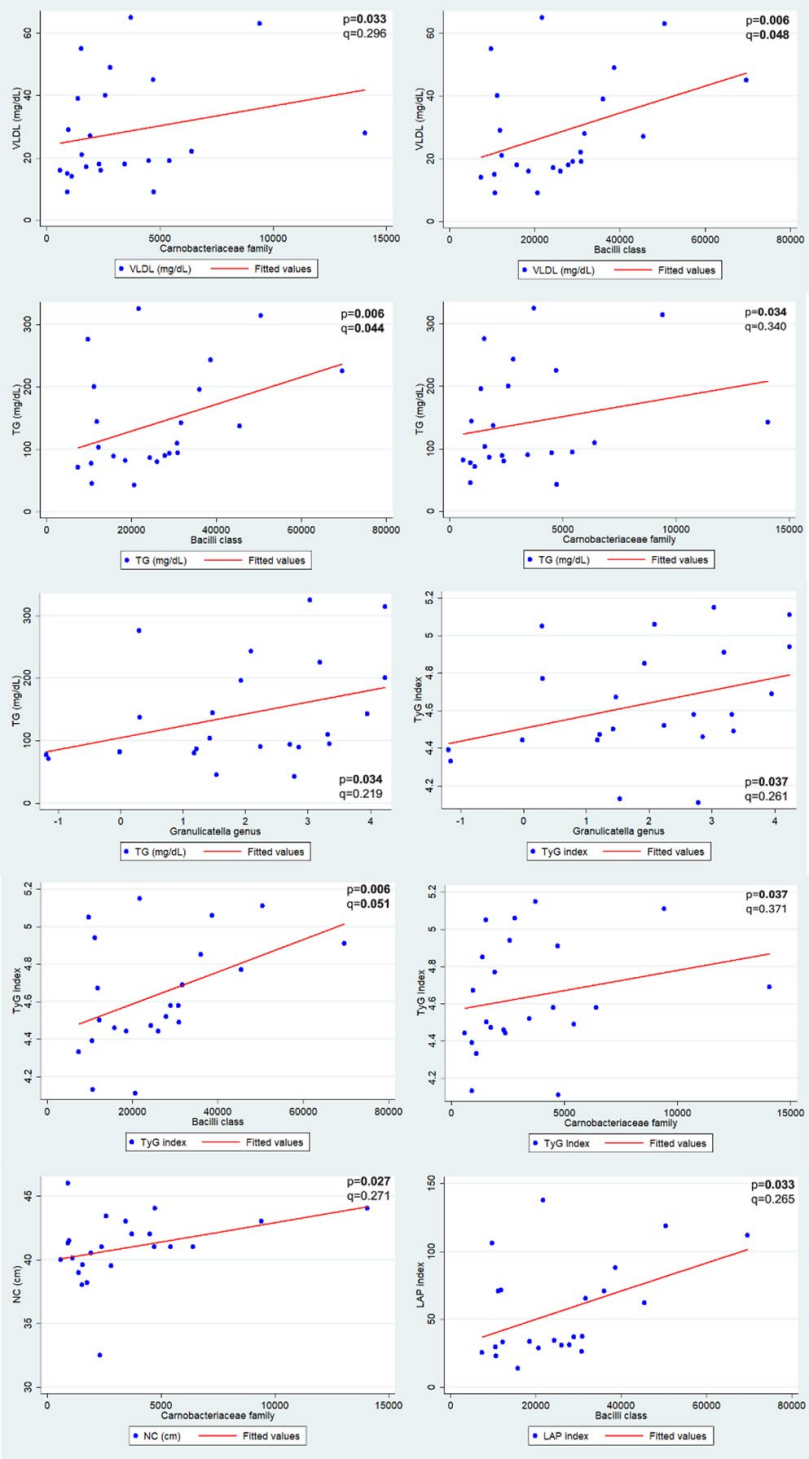
Association of the genus *Granulicatella*, class Bacilli and family Carnobacteriaceae found in greater abundance in women, with the cardiometabolic variables: TyG index, Triglycerides (TG), VLDL, Lipid Accumulation Product (LAP), and Neck Circumference (NC). Associations are shown with Spearman’s ρ (p) and Benjamini–Hochberg FDR-adjusted p-values (q)

The genus *Granulicatella* was associated with a higher serum level of triglycerides (rho = 0.444; *p* = 0.034) and a greater TyG index value (rho = 0.437; *p* = 0.037) in women. The family Carnobacteriaceae was associated with a higher neck circumference (rho = 0.459; *p* = 0.027), VLDL values (rho = 0.447; *p* = 0.033), triglycerides (rho = 0.444; *p* = 0.034) and TyG index (rho = 0.437; *p* = 0.037). The class Bacilli showed a positive correlation with LAP value (rho = 0.446; *p* = 0.033), TyG index (rho = 0.559; *p* = 0.006), VLDL (rho = 0.551; *p* = 0.006) and triglycerides (rho = 0.554; *p* = 0.006) (Fig. 3). After false discovery rate correction, only the class Bacilli remained significantly associated with cardiometabolic markers in women, showing positive correlations with triglyceride levels (rho = 0.554, q = 0.048), the TyG index (rho = 0.559, q = 0.044), and VLDL (rho = 0.551; q = 0.051). Associations involving the genera *Granulicatella* and the Carnobacteriaceae family were nominally significant but did not remain significant after FDR correction (q > 0.05) and should therefore be interpreted as exploratory (Fig. 3).

## Discussion

The salivary microbiota is crucial for maintaining oral and systemic health through complex host-microbe interactions [5, 6, 10, 11, 24]. Various studies have shown the association of the salivary microbiota profile with obesity, lifestyle habits, and cardiometabolic diseases [5, 6, 24, 25]. However, there is a lack of studies that analyzes the relationship between salivary microbiota and sex differences in the context of metabolic health. To our knowledge, this is the first study that explores the profile of salivary microbiota between men and women with excess body weight in Brazil. The main findings revealed that men showed greater alpha diversity compared to women at the family level. Furthermore, women exhibited a higher abundance of taxa previously reported in studies involving inflammatory or metabolic conditions, acknowledging that their functional role may vary according to oral health context.

In the present study, no difference in alpha diversity between sexes was observed at species and genus level, however, at the family level, greater alpha diversity was noted in men according to the Shannon and Simpson indices, emphasizing a greater balance and variety of bacterial families in this group. These findings are partially in line with those reported by Andrade et al. [12], who observed higher salivary microbial richness and diversity in male adolescents. Although sex differences were not the primary focus of that study and were evaluated in a younger population, the direction of the findings supports the notion that sex-related variation in salivary microbiota diversity may occur. Additionally, similar to our results, Andrade et al. [12] reported no significant differences in beta diversity between sexes.

The genera *Campylobacter*, *Granulicatella*, *Moryella*, and *Scardovia* were found in greater abundance in women. These findings align with Liu et al. [13], who specifically investigated sex differences in the oral microbiome using sex-stratified metagenomic analyses and reported differential abundance of a higher concentration of *Granulicatella* in Chinese women, conversely, the highest concentration of *Campylobacte*r was observed in men [13]. The genus *Campylobacter* is associated with cases of periodontitis, increased risk for the development of esophageal cancer, and cardiovascular diseases [26, 27, 28]. The putative species-level taxon *Campylobacter concisus*, which was also found in greater abundance in women, has been previously associated with inflammatory bowel diseases and the induction of pro-inflammatory cytokines, which enhance its invasion into host cells [29]. The greater abundance of the genera *Moryella* and *Scardovia* has been previously associated with the development of cavities and gastroesophageal reflux disease, since the abundance of these bacteria enhances gastric reflux [30–34]. Although the genera *Granulicatella* is part of the normal oral microbiota, it has been found in higher abundance in individuals with metabolic syndrome, suggesting a link to a pro-inflammatory environment [35]. In our study, this genus showed an association with higher triglyceride levels and an elevated TyG index in women. These findings suggest a potential link between *Granulicatella* land markers of metabolic imbalance, however, given the cross-sectional and exploratory nature of the analysis, no causal inferences can be made. Its greater presence may reflect systemic conditions related to lifestyle and oral dysbiosis. Notably, women had higher total body fat and lower muscle mass than men, which may have contributed to the increased abundance of this bacterium.

An important factor that may influence the composition of the salivary microbiota and partially explain some of the observed associations is periodontal health status. Periodontitis is a chronic inflammatory disease strongly associated with shifts in the oral microbiome, particularly characterized by increased abundance of anaerobic and proteolytic taxa and reduced microbial homeostasis. Evidence indicates that the composition of the subgingival microbiota varies according to the stage and severity of periodontitis, with more advanced stages showing greater dysbiosis and enrichment of pathogenic communities, regardless of geographic context [36]. Although saliva reflects a broader oral ecosystem, microbial signatures associated with periodontal inflammation may also be captured in salivary samples, potentially influencing observed associations with metabolic markers. Moreover, obesity itself has been shown to modulate the subgingival microbiota and to interact with periodontal status. A systematic review demonstrated that individuals with obesity present distinct subgingival microbial profiles compared to normal-weight individuals, particularly in the presence of periodontal disease, suggesting a synergistic relationship between excess adiposity, inflammation, and oral dysbiosis [37]. This interaction is especially relevant in the context of the present study, which included only individuals with excess body weight. Periodontal disease has also been linked to systemic metabolic alterations, including dyslipidemia. Previous studies have reported associations between periodontitis and increased plasma levels of cholesterol and triglycerides, reinforcing the concept of oral-systemic crosstalk mediated by chronic inflammation [38]. Thus, some of the associations observed between salivary taxa and cardiometabolic markers in the present study may reflect, at least in part, underlying periodontal conditions that were not clinically assessed.

It was also observed that the families Campylobacteraceae and Carnobacteriaceae, as well as the classes Campylobacteria and Bacilli, were more abundant in women. The class Bacilli has been previously associated with oral infections and periodontitis, foodborne illnesses, and severe antibiotic-resistant infections [39, 40]. In the present exploratory analysis, the abundance of the *Bacilli* class showed positive correlations with lipid-related cardiometabolic markers, including LAP, the TyG index, and circulating levels of VLDL and triglycerides in women. After correction for multiple testing, the associations with triglycerides, VLDL and the TyG index remained significant at an FDR threshold of 0.10, whereas the remaining correlations were nominal. These findings suggest a potential link between *Bacilli* abundance and adverse metabolic profiles in female participants, without implying independent or causal relationships. The family Carnobacteriaceae, from the phylum Bacillota (formerly Firmicutes), has been linked to obesity [41] and associated with increased cardiovascular risk and impaired lipid metabolism [42, 43]. Although its pathogenicity remains unclear, cases of infections and sepsis caused by its bacteria have been reported in humans [44]. In our study, higher abundance of Carnobacteriaceae in women was associated with larger neck circumference, elevated VLDL and triglycerides levels, and a higher TyG Index. These associations did not remain significant after FDR correction and should therefore be interpreted as hypothesis-generating. Additionally, they may reflect underlying oral health conditions not assessed in the present study, which could contribute to both microbial composition and metabolic traits [42, 43].

Sex-associated differences in the oral microbiota observed in the present study may be partially influenced by hormonal fluctuations across the menstrual cycle, however, this interpretation remains speculative, as no data on menstrual phase or circulating hormone levels were collected. Previous studies suggest that hormonal changes throughout the menstrual cycle can influence salivary flow, pH, inflammatory mediators, and the relative abundance of specific oral taxa, leading to transient shifts in oral microbial composition [44–48]. In this context, variations across menstrual phases have been associated with changes in alpha and beta diversity and with phase-specific differences in the abundance of genera such as *Streptococcus*, *Prevotella*, *Porphyromonas*, *Treponema*, *Campylobacter*, *Haemophilus*, *and Oribacterium* [46–48]. Nevertheless, given the absence of menstrual cycle phase information in our study, these mechanisms cannot be directly evaluated in the current analysis and should be interpreted with caution. Future studies specifically designed to capture hormonal status and menstrual phase are needed to clarify the role of female sex hormones in shaping the oral microbiome.

In contrast, male participants exhibited a distinct salivary microbial profile characterized by a higher abundance of certain taxa, such as those from the genera *Tannerella* and L*achnoanaerobaculum*. Although the absence of clinical oral assessment precludes conclusions regarding oral health status. The higher abundance of *Lachnoanaerobaculum* supports the findings of Ortiz et al. [14], who specifically investigated sex-specific differences in the salivary microbiome, although in a pediatric population with active dental caries. Notably, differences in the direction of association were observed for other taxa. While Ortiz and collaborators [14] reported higher abundances of *Actinomyces*, *Cardiobacterium*, and *Tannerella* in girls, these genera were more abundant in men in the present study. Such discrepancies may reflect differences in age, oral health status, disease context, and analytical resolution (species vs. genus level) between studies. Several of these taxa have been associated with oral health and lower intake of added sugars [49, 50]. However, in the present study, added sugar intake was not associated with microbial abundance, suggesting that other host-related factors may underlie the observed sex-specific patterns. Furthermore, the putative species-level assignment corresponding to *Tannerella serpentiformis*, found in greater abundance among the male participants of this study, has been previously linked to good oral health as this bacterium is related to the growth of protective biofilms, serving as an important mechanism for maintaining a symbiotic relationship between the oral microbiome and the immune system [50]. Additionally, the abundance of the genera *Lachnoanaerobaculum* was observed by Fan et al. [49] in participants with low consumption of added sugars, highlighting the impact of a balanced diet on the composition of the microbial profile [49]. On the other hand, a greater abundance of some the genera *Actinomyces* was found in greater abundance in men, although this genus is part of the normal oral microbiota, its higher abundance may be associated with oral infections, periodontitis, and can cause actinomycosis, a chronic infection that can affect the oral cavity under conditions of systemic stress [51, 52]. Moreover, the abundance of the phylum Actinobacteria, to which *Actinomyces* belongs, has been observed in individuals with obesity, which may indicate a potential relationship between this bacterial group and metabolic or inflammatory changes. The abundance of the phylum Actinobacteria has been previously observed in individuals with obesity [51]. The class Clostridia was also found in greater abundance in men, this class can act both as a pathogen and as a commensal in the gastrointestinal tract [53]. For example, the species *Clostridium difficile* is an important pathogen, responsible for antibiotic-associated diarrhea and pseudomembranous colitis [52].

The difference in microbial profiles between men and women may be related not only to hormonal differences between the sexes [13, 54], but also to differences in body composition [5, 6, 54]. Although the present study did not observe an association between bacteria and body composition across sexes, women had higher total body fat and gynoid fat values compared to men, while men presented a greater amount of total muscle mass. There currently a lack of studies linking salivary microbiota composition with muscle mass and body fat distribution between sexes, however, studies on gut microbiota show the influence of gynoid and android fat, as well as muscle mass, on microbiota composition [54–57]. Therefore, it is suggested that the differences in body composition between men and women may potentially impact the microbial profile, however, more studies are needed to investigate this relationship.

The identification of sex-specific microbial patterns in saliva is not only biologically relevant but also clinically meaningful. Given that salivary microbiota can be easily collected and reflects both oral and systemic health [5, 6], sex-related microbial profiles may represent candidate markers for future investigation as potential indicators of metabolic risk, pending confirmation in studies specifically designed to address sex differences. For policymakers and health practitioners, recognizing sex as biological variable in oral-systemic intersections reinforces the need for differentiated prevention and treatment strategies in obesity managements programs [57]. Ultimately, understanding these differences may guide the development of personalized nutritional or probiotic interventions that account for sex-specific microbial and metabolic responses.

A strength of the present study is the novel information addressed and found regarding the differences in salivary microbiota composition between overweight men and women and the association with cardiometabolic parameters, allowing for the development of new and individualized therapeutic strategies that consider sexual and metabolic factors. Another strength is the secondary analysis of data that were rigorously collected in a randomized controlled clinical trial, which took into account the matching between individuals, as well as a careful process for participant inclusion to control for the main factors that could be considered confounding variables in microbiota analysis, such as physical activity, alcohol consumption, smoking, and medication use. Among the limitations of the present study is the relatively small sample size, which was originally calculated for a clinical trial with different primary outcomes and not specifically designed to detect differences in oral microbiome composition. Moreover, the absence of a dental professional in the research team precluded a formal evaluation of oral health. Consequently, the study did not include the assessment of periodontal status or surrogate indicators of periodontitis, such as number of teeth, tooth mobility, or tooth loss due to loosening. As periodontal disease is known to influence the composition of the oral microbiome, this aspect should be considered when interpreting the results. Another limitation is the lack of information on menstrual cycle phase and oral contraceptive use at the time of sample collection, making it impossible to relate the obtained results to the hormonal fluctuations of the participants. Furthermore, the primary aim of this exploratory analysis was to investigate whether intrinsic sex-specific characteristics, including body composition and cardiometabolic profile, are associated with differences in salivary microbiota. Men and women in this study differed in some of these markers (e.g. body fat mass and muscle mass), which may partly drive the observed microbiota patterns. Due to the exploratory cross-sectional design and limited sample size, multivariable models adjusting for these factors were not performed to avoid overfitting. Future studies with larger samples and appropriate multivariable designs are needed to disentangle the independent contributions of sex and metabolic phenotype to salivary microbiota composition.

## Conclusion

In this exploratory cross-sectional secondary analysis, sex-related differences in salivary microbiota composition were observed among adults with excess body weight. Women showed a higher relative abundance of specific taxa, including the genera *Granulicatella* and *Moryella*, the class Bacilli, and the family Carnobacteriaceae, which have been previously associated with inflammatory and metabolic profiles. Men exhibited higher alpha diversity at the family level, however, differences in microbial composition were modest and not consistently observed across all taxonomic levels. Given the exploratory nature of the analyses and the potential influence of body composition and metabolic characteristics, these findings should be interpreted with caution and do not allow causal inferences regarding sex-specific microbial effects on cardiometabolic health. Nonetheless, the results support the relevance of considering sex as a biological variable in studies of the oral microbiome. Future studies specifically designed and powered to investigate sex-related differences, with appropriate adjustment for metabolic and body composition factors, are warranted to clarify these associations.

## Data Availability

Raw 16 S rRNA gene sequencing data from saliva samples have been deposited in the European Nucleotide Archive (ENA) under BioProject accession number PRJEB108444. The data are currently under embargo and will be made publicly available upon publication of the manuscript. Other data that support the findings of this study, including participant-related clinical and demographic information, contain sensitive human data and are available from the corresponding author upon reasonable request and under appropriate data-sharing agreements to ensure participant privacy.
